# Ultrasonographic evaluation of diastasis recti abdominis and its association with pelvic floor dysfunction in postpartum women: a cross-sectional study of a two-year retrospective cohort

**DOI:** 10.3389/fmed.2024.1441127

**Published:** 2024-12-05

**Authors:** Manrong Li, Nana Wang, Rui Wang, Bingyin Liang

**Affiliations:** ^1^Department of Rehabilitation, Shanghai East Hospital, Tongji University, Shanghai, China; ^2^Department of Rehabilitation, Shanghai Xinqidian Rehabilitation Hospital, Shanghai, China

**Keywords:** diastasis recti abdominis, ultrasonography, postpartum, risk factors, cross-sectional study, clinical characteristics

## Abstract

**Objective:**

This study aims to evaluate the ultrasonographic findings of Diastasis Recti Abdominis (DRA) in postpartum women, explore associated risk factors, and assess the relationship between DRA and pelvic floor dysfunction.

**Methods:**

This retrospective cross-sectional study included 143 parturient women diagnosed with DRA at our institution from January 2022 to December 2023. The study aimed to assess the ultrasonographic characteristics and clinical implications of DRA in postpartum women. The study participants were aged 21–43 years, carried a single fetus to term without instrumental delivery, and had no prior abdominal surgery. We excluded incomplete records and cases with congenital lumbar spine abnormalities or significant visceral disease. Ultrasound was employed to evaluate the DRA features, specifically the condition of the abdominal midline and echo types. A control group comprised 57 women who had normal deliveries without DRA. The primary outcome of this study was the measurement of rectus abdominis separation using ultrasonography. Secondary outcomes included the assessment of pelvic floor muscle function via electromyography (EMG), evaluation of pain levels, and the identification of risk factors associated with diastasis recti.

**Results:**

In the observation group, ultrasound revealed widened abdominal midlines in 122 cases (85.31%), discontinuity in 21 cases (14.68%), and various echo types indicating unequal, narrow band-like moderate, and slightly strong fibrous echoes. Significant differences were found in rectus abdominis separation and levator ani muscle hiatus area, with both being larger in the observation group compared to the control group (*p* < 0.05). Advanced maternal age, higher parity, higher pre-pregnancy BMI, and cesarean delivery were identified as significant risk factors for DRA occurrence in the logistic regression analysis.

**Conclusion:**

DRA is prevalent among postpartum women with specific ultrasonographic profiles indicating considerable abdominal muscle separation. The study highlights the importance of ultrasound in diagnosing DRA and suggests that certain demographic and delivery method factors increase the risk of this condition. These findings could guide clinicians in early diagnosis and intervention, potentially improving outcomes for affected women.

## Introduction

Diastasis Recti Abdominis (DRA) is characterized by the separation of the rectus abdominis muscles along the linea alba, a condition that frequently manifests in postpartum women (1). The prevalence of DRA varies widely but is notably higher among pregnant and postpartum populations due to the increased abdominal pressure from the growing uterus (2). Barbosa et al. (3) established a reference for the normal width of the linea alba in cadavers, offering essential baseline data for defining rectus diastasis, which can help contextualize the measurements obtained in clinical ultrasound assessments of DRA. Key risk factors associated with the development of DRA include multiple pregnancies, advanced maternal age, and certain delivery methods such as cesarean sections, which may predispose the abdominal musculature to separation. Clinically, if left untreated, DRA can lead to significant complications including weakened abdominal strength, chronic lower back and pelvic pain (4). Moreover, the instability in the abdominal core often correlates with pelvic floor dysfunctions, further complicating postpartum recovery (5). Recognizing these implications is crucial for timely and effective management of this widespread condition.

The assessment of DRA has traditionally relied on physical examination, but ultrasound has emerged as a superior diagnostic tool due to its precision and reliability (6). As demonstrated by Mendes et al., ultrasonography is an accurate method for measuring rectus abdominis muscle diastasis, particularly at the supra-umbilical and umbilical levels, providing reliable data for the assessment of muscle separation in clinical settings (7). Ultrasound provides a clear visualization of the linea alba, allowing clinicians to measure the exact degree of muscle separation, which is crucial for accurate diagnosis and treatment planning (8). Despite its advantages, current research on the ultrasound characteristics of DRA is limited, with few studies detailing the specific echo patterns associated with different severities of muscle separation. Moreover, the clinical relevance of these ultrasound findings is not fully established, highlighting a gap in linking ultrasonographic data with patient symptoms and long-term outcomes. This limitation underscores the necessity for more focused research that could validate ultrasound criteria and refine diagnostic protocols. The need for comprehensive research into the pathophysiology and clinical trajectory of DRA is imperative, particularly through the lens of ultrasound imaging. While the mechanical aspects of DRA, such as the role of increased intra-abdominal pressure and the stretching of the linea alba during pregnancy, are somewhat understood (9), the physiological underpinnings and progression of the condition postpartum remain poorly delineated.

This study aims to bridge these gaps by systematically documenting the ultrasonographic characteristics of DRA in a postpartum population and correlating these features with clinical outcomes. Objectives are to establish a clear ultrasound profile of DRA that can aid in the early diagnosis and to understand how these physical changes impact the health and recovery of affected women.

## Methods

### Study design and setting

This was a retrospective, cross-sectional study conducted at Shanghai East Hospital, involving a cohort of women who received prenatal care and delivery services at our institution between January 2019 and December 2021. Participants were identified based on clinical records, and DRA diagnosis was confirmed using ultrasonographic assessment. The study was designed to explore the ultrasonographic characteristics of DRA and its clinical implications in postpartum women. Ethical compliance was further ensured by obtaining informed consent from all participants, who were adequately informed about the aims of the study, the procedures involved, potential risks, and their rights to confidentiality and voluntary participation.

### Study participants

Potential participants were identified from women who attended the hospital for delivery between January 2022 and December 2023. Inclusion criteria were women aged 21–43 years with single, term pregnancies, no history of abdominal surgery, no congenital lumbar spine abnormalities, and no significant visceral diseases. Women with conditions such as severe gastrointestinal disorders (e.g., Crohn’s disease or advanced liver cirrhosis), chronic inflammatory conditions (e.g., rheumatoid arthritis with systemic involvement), or end-stage renal disease were excluded to avoid confounding factors in the assessment of DRA. Additional exclusion criteria included women with incomplete clinical data or preexisting abdominal wall disorders unrelated to pregnancy. From the eligible group, 143 women were included in the observational group, diagnosed with DRA based on ultrasound findings. The control group consisted of 57 women who had normal deliveries and did not exhibit signs of DRA, matched by age and gestational conditions. Final sample sizes for both groups were included in the analysis. Participants’ physical activity levels were not systematically recorded in this study. Future research should consider including this parameter, as it may influence abdominal muscle strength and the ultrasonographic measurements of rectus abdominis separation.

DRA was diagnosed using a combination of ultrasonographic measurements and clinical symptoms. Ultrasonography was employed to assess the inter-recti distance (IRD) at three anatomical landmarks: 3 cm above the umbilicus, at the umbilicus, and 3 cm below the umbilicus. A separation greater than 2 cm at any of these points served as the diagnostic threshold for DRA. In addition to increased IRD, the diagnosis also took into account clinical symptoms commonly associated with DRA, including abdominal muscle weakness, lower back pain, and pelvic floor dysfunction. Ultrasonographic measurements were conducted with the participant in a supine position to ensure consistency in evaluation. The diagnosis was confirmed by a certified radiologist, who reviewed both the increased IRD and the presence of the associated clinical symptoms.

The control group consisted of women who did not exhibit signs of DRA, defined as: (1) a maximum inter-recti distance of less than 2 cm, as measured by ultrasonography at any point along the linea alba; and (2) no visible or palpable separation of the rectus abdominis muscles during a standardized physical examination conducted by a trained clinician.

### Data collection

#### Variables and outcome measures

The primary outcome measures for this study were the measurement of the rectus abdominis separation at three defined anatomical points and the area of the levator ani muscle hiatus, as visualized on ultrasound. A US examination of the abdomen was performed with an Epiq 7 (Philips) ultrasound machine equipped with both a micro-convex (frequency 5–8 MHz) and a high-frequency linear transducer (4–18 MHz). All ultrasound examinations were conducted by experienced sonographers in musculoskeletal imaging, ensuring high consistency and accuracy in the measurements. Standardized imaging protocols were followed, with the participants positioned in the supine position. The sonographers adjusted imaging settings, including depth, gain, and focus, to optimize image quality. Measurements were taken at three anatomical landmarks: 3 cm above the umbilicus, at the umbilicus, and 3 cm below the umbilicus. These measures are critical for assessing the severity and impact of DRA. Secondary outcomes included the assessment of pelvic floor muscle function using electromyography (EMG) and the evaluation of associated symptoms such as pelvic and lower back pain (10). These outcomes were quantified using validated pain and functional impairment scales during the postpartum follow-ups.

Initial data were collected during the postpartum hospital stay, typically within the first week following delivery. Follow-up data were subsequently collected at 6 weeks, 3 months, and 6 months postpartum to assess changes in DRA and related symptoms over time.

### Statistical analysis

Statistical analyses were performed using SPSS version 26.0. Descriptive statistics were used to summarize the demographic and clinical characteristics of the study groups. For between-group comparisons of continuous variables, independent t-tests were used for normally distributed data, and Mann–Whitney U tests were used for non-normally distributed data. Categorical variables were compared using the Chi-square test or Fisher’s exact test, as appropriate. To account for the possibility of Type I errors due to multiple comparisons, we performed Bonferroni correction for the comparisons between the two groups. The significance level was adjusted to *p* < 0.05/n, where n represents the number of comparisons made. For the multivariate analysis, logistic regression was conducted to assess the independent associations of potential risk factors with the presence of DRA, adjusting for confounding variables such as age, BMI, parity, and mode of delivery. The results were presented as odds ratios (OR) with 95% confidence intervals (CI).

## Results

### Ultrasonographic findings

This study conducted detailed ultrasonographic evaluations on 143 postpartum women diagnosed with DRA and compared these results to 57 control participants without DRA. The primary ultrasonographic findings demonstrated significant variations in rectus abdominis separation and the condition of the abdominal midline within the study cohort. In the observational group, a notably high prevalence of DRA-related abnormalities was detected: Widened abdominal midline: Observed in 122 participants (85.31%), indicating a substantial alteration in the linea alba’s width.

Discontinuity of the linea alba: Present in 21 cases (14.68%), which typically signifies a severe separation. Echo types: Displayed considerable variation with 25 cases (17.48%) having unequal echoes, 60 cases (41.95%) with narrow band-like moderate echoes, and 58 cases (40.55%) showing slightly strong fibrous echoes. The mean rectus abdominis separation at the umbilicus was 28.51 ± 3.87 mm for the observational group, significantly larger than the 24.05 ± 4.63 mm observed in the control group (*p* < 0.05; Table 1). These differences not only underscore the diagnostic criteria of DRA but also highlight the increased severity of the condition in the observational group compared to the controls. Statistical analysis reinforced these findings, revealing a higher incidence and broader spectrum of ultrasonographic features of DRA in the observational group. The significant variation in echo types and the condition of the abdominal midline correlate strongly with the clinical manifestations of DRA, suggesting that ultrasonography is a critical tool in identifying and evaluating the severity of this condition in postpartum women.

**Table 1:** Ultrasound characteristics of DRA in the observational group of parturient women.

Ultrasound characteristics		Cases	Composition ratio (%)
Condition of the abdominal midline	Widened abdominal midline	122	85.31
	Discontinuity of abdominal midline continuity	21	14.68
Echo types	Unequal echoes narrow band-like	25	17.48
	Moderate echoes	60	41.95
	Slightly strong fibrous echoes	58	40.55

DRA is defined as the pathological widening of the gap between the two rectus abdominis muscles, often involving the stretching of the connective tissue (linea alba) at the midline. This separation is measured by the distance between the two muscles at specific points along the midline and is typically considered clinically significant when the gap exceeds 2 cm. The width of the linea alba, on the other hand, refers to the measurement of the connective tissue itself, without reference to the muscular separation. While an increased width of the linea alba is commonly observed in individuals with DRA, it is important to differentiate between the measurement of the connective tissue width and the actual separation of the muscles.

### Clinical data and comparative analysis

The study collected extensive clinical and demographic data from all participants, focusing on key metrics such as age, pre-pregnancy Body Mass Index (BMI), medical history, and delivery methods. The observation group, consisting of women diagnosed with DRA, had an average age of 30.34 ± 2.21 years, which was significantly older than the 28.83 ± 2.46 years recorded for the control group. Notably, the preferred mode of delivery differed between the groups; cesarean sections were predominant in the observation group (61.54%), whereas vaginal deliveries were more common in the control group (71.93%). Quantitative measurements of rectus abdominis separation at three anatomical landmarks—3 cm above the umbilicus, at the umbilicus, and 3 cm below the umbilicus—revealed greater separations in the observation group across all points. Specifically, the average separation at the umbilicus was 28.51 ± 3.87 mm for the observation group compared to 24.05 ± 4.63 mm in the control group. The levator ani muscle hiatus area also showed significant differences, with the observation group recording a larger mean area of 24.68 ± 3.17 cm^2^ versus 16.15 ± 2.85 cm^2^ in the control group. These findings are graphically summarized in Table 2, which illustrate the disparities in clinical indicators between the groups. These statistical outcomes emphasize the strong association between the clinical manifestations of DRA and increased metrics of rectus abdominis separation and levator ani muscle hiatus area. The observed variations in clinical indicators underscore the impact of delivery method and demographic factors on the severity of DRA, providing essential insights into its pathophysiology and potential preventive strategies.

**Table 2:** Comparison of clinical indicators between the two groups of parturient women.

Group	*n*	Rectus abdominis separation (mm)			Levator ani muscle
		3 cm above the umbilicus	At the umbilicus 3 cm	Below the umbilicus	Hiatus area (cm^2^)
Control group	57	24.05 ± 4.63	20.36 ± 3.41	19.97 ± 3.78	16.15 ± 2.85
Observation group	143	28.51 ± 3.87	29.98 ± 4.35	23.56 ± 4.83	24.68 ± 3.17
*t*		6.051	11.567	4.975	13.347
*p*		0.000	0.000	0.000	0.000

### Analysis of risk factors

Initial univariate analysis highlighted several variables that might increase the risk of DRA among postpartum women. Notably, advanced age (≥35 years), multiple births, a pre-pregnancy BMI of 25 kg/m^2^ or more, and cesarean section emerged as potential risk factors, each showing preliminary associations with DRA with *p*-values less than 0.10. These findings guided the selection of variables for more rigorous scrutiny through multivariate logistic regression to control for confounding influences. The multivariate analysis robustly confirmed the significant role of these factors in predicting DRA. Specifically, women aged 35 years or older were found to be over three times more likely to develop DRA compared to younger women (OR = 3.206, 95% CI: 2.014–5.525, *p* = 0.002). Similarly, undergoing a cesarean section nearly tripled the risk (OR = 3.078, 95% CI: 1.633–2.188, *p* < 0.05), highlighting the impact of surgical delivery on abdominal muscle integrity. Multiple births also increased the likelihood of experiencing DRA (OR = 2.436, 95% CI: 2.316–3.765, *p* < 0.001), possibly due to enhanced mechanical pressure and stretching of the abdominal wall. Additionally, a higher pre-pregnancy BMI was associated with a modest increase in risk (OR = 1.513, 95% CI: 1.021–1.307, *p* < 0.05), underscoring the influence of maternal body mass on abdominal structure stress. These statistically significant risk factors not only elucidate the epidemiological aspects of DRA but also offer actionable insights. The strong associations suggest that interventions such as managing body weight, considering the risks of multiple pregnancies, and evaluating the necessity of cesarean sections could be beneficial in reducing the incidence of DRA (Table 3). This approach could inform targeted preventive strategies in clinical settings, aimed at minimizing the occurrence of DRA among at-risk groups.

**Table 3:** Analysis of risk factors for postpartum DRA.

Factors	*b*	Standard error	wald	*p*	Exp(B)
Age ≥ 35 years	1.767	0.266	8.374	0.002	3.206 (2.014,5.525)
Parity	1.112	0.156	7.416	0.000	2.436 (2.316,3.765)
Maternal pre-pregnancy BMI	0.035	0.026	3.527	0.041	1.513 (1.021,1.307)
Mode of delivery	1.346	0.133	4.118	0.025	3.078 (1.633,2.188)
Rapid contraction EMG value	0.005	0.022	0.245	0.621	1.005 (0.984,1.027)
Tense contraction EMG value	0.027	0.018	1.504	0.220	1.025 (0.984,1.077)
Constant	−2.038	0.525	8.236	0.019	0.223

### Secondary outcomes and associated symptoms

The study utilized electromyography (EMG) to measure the function of pelvic floor muscles among postpartum women, revealing significant functional discrepancies between those with and without DRA (Table 4). Specifically, the EMG results for rapid and tense contractions were notably lower in the observation group, which included women diagnosed with DRA. This group registered mean EMG values of 39.48 μV for rapid contraction and 30.62 μV for tense contraction, compared to 43.21 μV and 32.43 μV in the control group, respectively, with these differences achieving statistical significance (*p* < 0.05). Moreover, the observation group reported higher levels of pelvic and back pain, with average pain scores of 6.2 on a 10-point scale, significantly higher than the 3.5 reported by the control group. These findings not only underscore the decreased pelvic floor muscle function in women with DRA but also illustrate the condition’s extensive impact on overall musculoskeletal and nerve function. The correlation between weakened pelvic floor muscles, increased pain, and the presence of DRA highlights critical areas for postpartum care. These insights suggest that comprehensive management strategies extending beyond the mere monitoring of DRA progression should be implemented. Interventions should include targeted physical therapy aimed at strengthening both the abdominal and pelvic floor muscles. Such therapeutic strategies are likely to alleviate pain symptoms and enhance overall quality of life. By adopting a multidisciplinary approach to postpartum rehabilitation, these interventions could significantly reduce the long-term health complications associated with DRA, potentially improving functional outcomes for affected women. This holistic care model not only addresses immediate physical issues but also contributes to better long-term health prospects for postpartum women facing challenges related to DRA.

**Table 4:** Comparison of clinical data between two groups of parturients.

Factors	Control group (*n* = 57)	Observation group (*n* = 143)	*χ*^2^/*t*	*p*
Age	30.34 ± 2.21	28.83 ± 2.46	5.446	0.001
Age ≥ 35 years	4 (7.01)	42 (29.37)	7.687	0.005
Parity	0.48 ± 0.15	0.92 ± 0.21	15.675	0.000
Birth weight of newborn (g)	3,023 ± 251.42	3,267 ± 263.18	5.512	0.007
Maternal pre-pregnancy BMI (kg/m^2^)	25.43 ± 2.35	26.91 ± 2.18	4.83	0.00
Mode of delivery				
Vaginal delivery	41	55	15.001	0.000
Cesarean section	16	88		
Education				
Below Bachelor’s degree	36 (63.15)	78 (54.54)	1.023	0.278
Bachelor’s degree and above	21 (36.84)	65 (45.45)		
Resting membrane potential	4.13 (2.75,7.08)	4.38 (3.13,6.85)	0.956	0.461
Rapid contraction EMG value	39.48 (29.18,51.74)	43.21 (32.86,61.55)	3.267	0.001
Tense contraction EMG value	30.62 (20.83,35.86)	32.43 (25.21,44.29)	2.562	0.011
Endurance contraction EMG value	26.37 (20.20,33.27)	27.07 (21.11,40.10)	1.902	0.057

## Discussion

This study revealed that DRA in postpartum women is associated with increased rectus abdominis separation and levator ani muscle hiatus, exacerbated by risk factors such as advanced age, higher BMI, multiple births, and cesarean delivery. These conditions correlate with weakened pelvic floor muscles and increased pain, underscoring the importance of targeted postpartum rehabilitation.

### Interpretation of main findings

The ultrasonographic assessment in this study revealed significant findings, including a widened abdominal midline and various echo types such as unequal, narrow band-like moderate, and slightly strong fibrous echoes, which are critical markers for diagnosing DRA (11). These features align with previous research, such as that by A M Rath et al. (12), which also highlighted the prevalence of echo pattern variability in patients with DRA and its potential diagnostic value. The clinical relevance of these ultrasonographic measures is profound; the width of the rectus abdominis separation and the condition of the levator ani muscle hiatus captured via ultrasound are strongly correlated with the functional impairments and symptomatic severity observed in DRA patients (13). These findings suggest that ultrasound, as a non-invasive and readily available diagnostic tool, should be integral to the postpartum evaluation process, aiding in the early detection and stratification of DRA severity, thereby facilitating timely and appropriate therapeutic interventions.

### Risk factors and their implications

The identification of risk factors such as advanced maternal age, higher BMI, increased parity, and cesarean delivery as significant predictors of DRA is consistent with the broader body of research on this condition. Studies like those have similarly reported that these factors contribute to the mechanical and hormonal stresses that predispose the abdominal musculature to separation (14). Advanced age and higher BMI, for example, are known to affect tissue elasticity and load-bearing capacity, which can exacerbate the separation of the rectus abdominis muscles during and after pregnancy. The association with cesarean delivery might be attributed to surgical factors and the healing processes that follow, which may disrupt the normal integrity and function of the abdominal wall (15). Understanding these risk factors provides crucial insights for both prevention and management of DRA. Antenatal education can focus on modifiable risk factors, such as encouraging optimal weight management and discussing delivery options. For women at higher risk, proactive measures including targeted physical therapy might be recommended to strengthen the abdominal and pelvic floor muscles, potentially mitigating the severity of DRA post-delivery (16). Additionally, routine postpartum screening for DRA in women with these risk profiles could lead to earlier intervention and improved outcomes.

### Secondary outcomes

The study’s findings highlight a significant correlation between DRA and pelvic floor dysfunction, as well as increased pelvic and back pain. This association likely stems from the biomechanical changes in the abdominal and pelvic region due to the separation of the rectus abdominis muscles. The weakening of the abdominal wall can lead to an altered load distribution across the pelvic girdle, as supported by Yukti Jobanputtra, who noted that pelvic floor disorders in postpartum women are often accompanied by conditions like DRA that disrupt the integrity of the musculoskeletal support system (13). These changes can increase the strain on the pelvic floor, exacerbating pain and dysfunction. Given the clear link between DRA and pelvic health, targeted rehabilitation programs are crucial. Physical therapy interventions focusing on strengthening both the abdominal and pelvic floor muscles can be effective in mitigating the symptoms of DRA and improving overall pelvic health (17). Techniques such as deep abdominal muscle training, pelvic tilts, and pelvic floor exercises have been shown to reduce the gap in the abdominal muscles and alleviate associated pelvic pain. Moreover, lifestyle modifications, including proper posture training and weight management, should be emphasized during the postpartum period to support the recovery process and prevent further deterioration of muscle function.

### Limitations and future research

#### Study limitations

While this study offers valuable insights into diastasis recti abdominis (DRA) and its implications, several limitations should be acknowledged. The retrospective design restricts the ability to establish causality between observed factors and the development of DRA. Additionally, selection biases may have influenced the findings, as participants were recruited from a single healthcare facility, which may affect the generalizability of the results. The reliance on ultrasound measurements and self-reported symptoms also introduces the potential for measurement variability and subjective bias, particularly in the reporting of pain and functional impairment. Furthermore, data collection was based on participants’ clinical histories, which could be incomplete or prone to missing information due to the nature of medical records. While efforts were made to ensure the accuracy and consistency of data, the possibility of inaccurate or incomplete data entry cannot be fully excluded. Another limitation is the single-hospital design, which may limit the broader applicability of the findings. The control group was exclusively composed of women who had vaginal deliveries, which introduces potential bias when compared to the observational group of women with DRA. Future studies should consider multi-center designs and include a wider variety of delivery types (including cesarean sections) in both observational and control groups. Such approaches would ensure a more balanced and comprehensive comparison, ultimately enhancing the generalizability of the findings.

#### Suggestions for future research

Future research should address these limitations and further advance the understanding of Diastasis Recti Abdominis (DRA). Establishing standardized diagnostic criteria for DRA is essential to ensure consistent identification and management of the condition across various clinical settings. Longitudinal studies that follow patients from the prenatal period through postpartum recovery would offer valuable insights into the natural progression of DRA and the effectiveness of different treatment strategies. Additionally, randomized controlled trials are necessary to evaluate the impact of specific interventions, such as various physical therapy modalities, on DRA resolution, pelvic floor function, and overall quality of life. These studies could provide the evidence needed to develop evidence-based guidelines for the prevention and treatment of DRA.

Moreover, future research could benefit from incorporating dynamic pelvic floor ultrasonography as an objective tool to assess pelvic floor muscle function. This would deepen our understanding of its relationship with DRA and associated symptoms. To further improve the objectivity of pain assessments, the integration of additional clinical tests or biomarkers for chronic pain is recommended. Tools such as pressure pain threshold testing or serum biomarkers for inflammation could provide objective measures of pain and muscle dysfunction, offering a more comprehensive assessment of symptoms and helping to validate the findings of this study.

## Conclusion

This study has detailed significant associations between DRA and various risk factors, including advanced maternal age, higher BMI, multiple births, and cesarean delivery. The ultrasonographic findings provide a reliable method for assessing the severity of DRA, which is linked with weakened pelvic floor muscles and increased discomfort. These insights are crucial for enhancing postpartum care by improving diagnostic accuracy and tailoring rehabilitation programs to better manage and potentially prevent DRA. The findings urge a reevaluation of current postpartum protocols to integrate routine screening for DRA and associated pelvic floor issues, ensuring early intervention. Healthcare providers are called to incorporate these insights into practice to optimize care for postpartum women, while researchers should continue to refine DRA diagnostics and treatment strategies through further rigorous studies.

## Data Availability

The original contributions presented in the study are included in the article/supplementary material, further inquiries can be directed to the corresponding author/s.
